# Extracellular Vesicles and Cell–Cell Communication in the Cornea

**DOI:** 10.1002/ar.24181

**Published:** 2019-06-10

**Authors:** James D. Zieske, Audrey E. K. Hutcheon, Xiaoqing Guo

**Affiliations:** ^1^ Department of Ophthalmology, Schepens Eye Research Institute/Massachusetts Eye and Ear Harvard Medical School Boston Massachusetts

**Keywords:** cornea, extracellular vesicles, corneal endothelium, cell–cell communication, stromal matrix, exosomes, nanovesicles

## Abstract

One question that has intrigued cell biologists for many years is, “How do cells interact to influence one another's activity?” The discovery of extracellular vesicles (EVs) and the fact that they carry cargo, which directs cells to undergo changes in morphology and gene expression, has revolutionized this field of research. Little is known regarding the role of EVs in the cornea; however, we have demonstrated that EVs isolated from corneal epithelial cells direct corneal keratocytes to initiate fibrosis. Intriguingly, our data suggest that EVs do not penetrate epithelial basement membrane (BM), perhaps providing a mechanism explaining the importance of BM in the lack of scarring in scrape wounds. Since over 100‐million people worldwide suffer from visual impairment as a result of corneal scarring, the role of EVs may be vital to understanding the mechanisms of wound repair. Therefore, we investigated EVs in *ex vivo* and *in vivo*‐like three‐dimensional cultures of human corneal cells using transmission electron microscopy. Some of the major findings were all three major cell types (epithelial, fibroblast, and endothelial cells) appear to release EVs, EVs can be identified using TEM, and EVs appeared to be involved in cell–cell communication. Interestingly, while our previous publication suggests that EVs do not penetrate the epithelial BM, it appears that EVs penetrate the much thicker endothelial BM (Descemet's membrane). These findings indicate the huge potential of EV research in the cornea and wound healing, and suggest that during homeostasis the endothelium and stromal cells are in communication. Anat Rec, 2019. © 2019 The Authors. *The Anatomical Record* published by Wiley Periodicals, Inc. on behalf of American Association of Anatomists.

Intercellular communication is essential to cell development and homeostasis in multicellular organisms. These communications can be localized or distant. Local communication involves direct contact between cells using structures, such as gap junctions, which allow cytoplasmic contents to be shared. Distant communication involves signaling between cell types that can be separated by short or long distances. A classical example is hormones, which can travel throughout the body via the blood stream. The communication may also be between cells in an organ, such as the epithelial cells and the underlying stromal keratocytes in the cornea. Recently, much of the interest in cell–cell communication revolves around EVs, which are membrane based and carry “cargo” (i.e., proteins, lipids, miRNA, mRNA, and DNA) that can affect the activity of the recipient cell (Di Rocco et al., 2016; Dos Anjos Pultz et al., 2017; French et al., 2017; Gopal et al., 2017; Ha et al., 2016; Kahlert and Kalluri, 2013; Maas et al., 2017). The EVs bind to the recipient cell through ligand receptor interactions, and either signal from the cell surface or are internalized to direct the recipient cell from within (French et al., 2017). Extracellular vesicles are generally divided into two groups—ectosomes and exosomes—based on size and method of formation and release. Ectosomes, also called microvesicles, range in size from 100 to 500 nm and are formed by the outward budding of the plasma membrane. Exosomes are smaller than ectosomes (30–100 nm) and are derived from endosomes. In this pathway, a portion of the plasma membrane is endocytosed, forming an endosome that is either degraded by the lysosomes or redirected and reloaded with cargo, and subsequently, released into the external environment. Since exosomes and ectosomes share overlapping sizes, which are not totally agreed upon, and functions, the scientific community has agreed to use “EV” to indicate all types of nanovesicles (Di Rocco et al., 2016; Dos Anjos Pultz et al., 2017; French et al., 2017; Gopal et al., 2017; Ha et al., 2016; Kahlert and Kalluri, 2013; Maas et al., 2017).

The presence of EVs has been known for many years; however, these vesicles were generally considered artifacts or a method to remove waste proteins (Johnstone, 2005). The term “exosomes” was first used by Trams et al. (1981), but it was not until the early 2000s, when a series of papers demonstrating that cancer cells release EVs, that interest in EVs became wide spread (Kharaziha et al., 2012). These manuscripts demonstrated that tumor cells release EVs that contain and transfer oncogenic cargo to host cells (Di Rocco et al., 2016; Dignat‐George and Boulanger, 2011; Dos Anjos Pultz et al., 2017; Gopal et al., 2017; Kahlert and Kalluri, 2013; Puddu et al., 2010; Quesenberry et al., 2015; Webber et al., 2010). Subsequently, EVs have been associated in the initiation, development, and prognosis of several types of cancer, including pancreatic, lung, breast, and prostrate (Di Rocco et al., 2016; Dos Anjos Pultz et al., 2017; French et al., 2017; Gopal et al., 2017; Ha et al., 2016; Kahlert and Kalluri, 2013; Maas et al., 2017). In many of these, the epithelial cells have become oncogenic and have begun to undergo epithelial–mesenchymal transition (EMT) (Gopal et al., 2017). As the cells undergo EMT, the cargo in the EVs is altered to stimulate the underlying fibroblasts to prepare a microenvironment that will promote tumor growth. The EVs from tumor cells contain oncogenic proteins and miRNAs that effect the transcription and/or translation of new proteins in target cells (Di Rocco et al., 2016; Dos Anjos Pultz et al., 2017; French et al., 2017; Gopal et al., 2017; Ha et al., 2016; Kahlert and Kalluri, 2013; Maas et al., 2017). One of the proteins frequently present in the cargo is TGF‐β1, which stimulates the fibroblasts to become myofibroblasts and secrete an altered matrix (Tan et al., 2016; Webber et al., 2010). Extracellular vesicles are also involved in metastasis (Di Rocco et al., 2016; Dos Anjos Pultz et al., 2017; French et al., 2017; Gopal et al., 2017; Ha et al., 2016; Kahlert and Kalluri, 2013; Maas et al., 2017), where the cancer EVs are released by the tumor, enter the bloodstream, and adhere to specific sites through ligand–receptor interactions, many of which are integrin dependent (French et al., 2017). We postulate that EVs are also involved in corneal wound repair. In this concept, EVs released from epithelial cells migrating to cover a wound would promote transformation of the stromal cells to myofibroblasts. We have demonstrated a portion of this concept in a cell culture model (Han et al., 2017), and parts of this concept have been observed in kidney and cutaneous injuries (Tan et al., 2016; Than et al., 2017).

While thousands of references for EVs are found in the literature, only very limited information is reported for the cornea. Azar and colleagues reported that EVs secreted by corneal fibroblasts could transport proteins to vascular endothelial cells demonstrating the important concept that cell–cell communication via EV is functional in the cornea (Han et al., 2015). In a second paper (Han et al., 2017), which was a collaboration between my lab and Dr. Azar's, we examined if EVs isolated from corneal epithelium interacted with corneal keratocytes/fibroblasts, and if these EVs stimulated fibrosis, as indicated by the expression of α‐smooth muscle actin (SMA). Important findings included that epithelial EVs interacted with keratocytes and stimulated SMA expression. The EVs appeared to primarily attach to the surface of the cells and no obvious internalization was seen. It is not clear if the EVs are functioning at the cell surface or if they are rapidly degraded when they are internalized, making them difficult to detect inside the cell.

In the current investigation, we examined if it was possible to localize EVs in an *ex vivo* cornea and in a 3D *in vivo*‐like coculture of human corneal cells.

## MATERIALS AND METHODS

### Isolate Cells for Culture

Human corneas were obtained from National Disease Research Interchange (NDRI, Philadelphia, PA) and selected based on published criteria (Joyce, 2005). All procedures and methods used in these studies adhered to the tenets of the Declaration of Helsinki, and the Schepens Eye Research Institute IRB deemed this research to be exempt.

### Human Corneal Endothelial Culture

Primary human corneal endothelial cells were isolated and cultivated, as previously described (Chen et al., 2001; Zhu and Joyce, 2004). In brief, after washing and treating the corneas with gentamicin and antibiotic/antimycotic solution, they were placed endothelial side up and the Descemet's membrane with endothelium was carefully removed and placed in Chen's medium (OptiMEM‐1 + 8% FBS, 5 ng/mL EGF, 20 ng/mL NGF, 100 μg/mL pituitary extract, 20 μg/mL ascorbic acid, 200 μg/mL calcium chloride, 0.08% chondroitin sulfate, 50 μg/mL gentamicin, and antibiotic‐antimycotic solution diluted 1:100) (Chen et al., 2001). The strips of tissue obtained were then centrifuged and treated with 0.02% EDTA solution. Finally, the endothelial cells were isolated from the Descement's membrane, resuspended in Chen's medium, and grown in a FNC‐coated 12‐well tissue culture plate.

### Human Corneal Fibroblasts Culture

Primary human corneal fibroblasts (HCFs) were isolated, as previously described in Guo et al. (2007) from the donor cornea, which was denuded of both the corneal endothelium (as described above) and epithelium (Guo et al., 2007). In brief, the stromal tissue was cut into small 2 × 2 mm pieces, placed in T25 flasks (4 or 5 pieces per well), and allowed to adhere. Explants were cultured with Eagle's Minimum Essential Medium (EMEM: ATCC; Manassas, VA) containing 10% fetal bovine serum (FBS: Atlantic Biologicals; Lawrenceville, CA). All cultures were cultivated for 1–2 weeks, passaged into 100 mm cell culture plates, grown to 100% confluence, and then used in the culture system. Passages up to number 6 were used throughout the experiments.

#### *3D culture*

HCF stromal constructs were assembled as previously described (Guo et al., 2007; Karamichos et al., 2010, 2011). Briefly, primary HCFs (10^6^ cells/mL) were plated on transwell inserts (Costar; Charlotte, NC) in EMEM plus 10% FBS and 0.5 μM 2‐O‐α‐d‐glucopyranosyl‐l‐ascorbic acid (VitC: Wako Chemical USA, Inc.; Richmond, VA) for 4 weeks. The cultures were either fixed and processed for transmission electron microscopy (TEM) or seeded with endothelial cells (5 × 10^6^ cells/mL) to produce a coculture. To cultivate the coculture, Chen's medium was added to the top (or inner) well, which was in contact with the endothelial cells, allowing them to grow in their preferred medium, while EMEM +10% FBS + 0.5 μM VitC was added to the outer well, which was in contact with the bottom of the construct, allowing the HCF to continue to grow in their preferred media. Cocultures were grown in this manner for an additional 5–7 days at 37°C, 5% CO_2_, after which they were fixed and processed for TEM.

#### *Rabbit corneas*

All studies were conducted in accordance with the NIH Guidelines for the Care and Use of Laboratory Animals, and approved by the Schepens Eye Research Institute/Massachusetts Eye and Ear IACUC. Briefly, an adult Dutch Belted rabbit (Covance, Inc.; Princeton, NJ) was euthanized with 120 mg/kg sodium pentobarbital (Euthasol) by IV injection through the marginal ear vein, and the corneas were removed and processed for TEM.

#### *Transmission electron microscopy*

At the designated time, the cocultures were fixed in ½ strength Karnovsky's fixative (2% paraformaldehyde, 2.5% gluteraldehyde in cacodylate buffer, pH 7.4) and processed for TEM using standard procedures, as described previously (Gipson et al., 1983). Briefly, constructs were rinsed in PBS, processed through postfixation in 2% osmium tetroxide, *en bloc* stained in 0.5% uranyl oxide, dehydrated with alcohol to propylene oxide, and embedded (Embed 812: Electron Microscopy Sciences; Hatfield, PA). Thin sections were cut transverse to the plane of the construct using a diamond knife on an ultramicrotome (LKB; Bromma, Sweden). The sections were viewed and photographed with an electron microscope (Tecnai G2 Spirit: FEI Company; Hillsboro, OR).

## RESULTS

**Figure 1:**
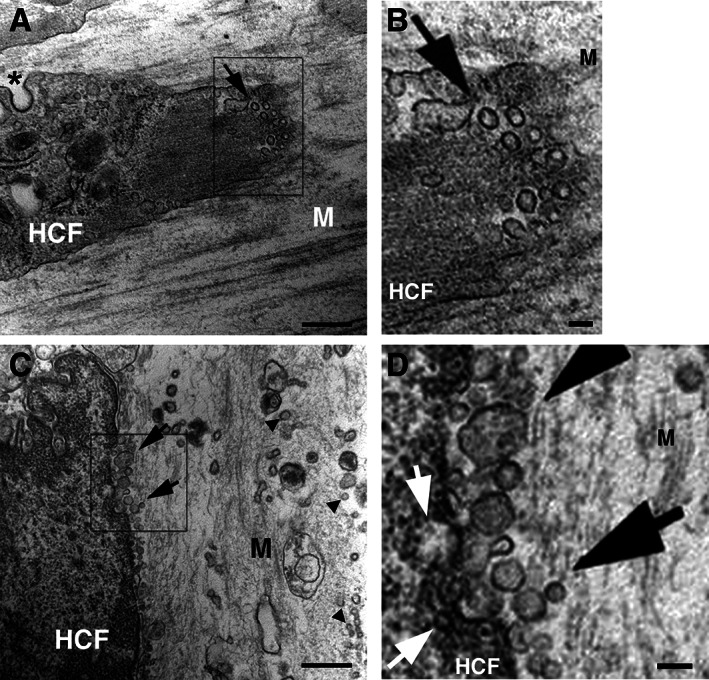
Transmission electron microscopy images showing that EVs are apparent in a 3D *in vivo*‐like culture. (**A** and **B**) EVs are present in the HCF (black arrow) at the tip of an area that appears to contain actin filaments. * indicates a pit in the HCF (A). (B) Enlarged area that is outlined in (A). (**C** and **D**) EVs of various sizes are apparent adjacent to the HCF (black arrows), within the matrix (C, arrowheads), and within the cell (D, white arrows). (D) Enlarged area that is outlined in (C). HCF , human corneal fibroblast; M, matrix; Bars: (A and C) = 500 nm; (B and D) = 100 nm.

We have previously demonstrated that EVs can be isolated from cultured corneal epithelial and stromal cells and identified by TEM (Han et al., 2017). To examine if the secretion of EVs can be visualized in a 3D *in vivo*‐like culture, HCFs were cultivated in a VitC‐rich medium that stimulates extracellular matrix secretion and assembly. In these cultures, EVs were apparent both in cells (Fig. 1A,B) and adjacent to cells (Fig. 1C,D), as indicated by black arrows. For better visualization of the EVs, the outlined areas in Figure 1A,C were enlarged to produce Figure 1B,D, respectively. As observed in Figure 1A,B, quite a few EVs were present in one area of the cell, which also appeared to contain actin filaments. Figure 1C,D showed large numbers of EVs of varying sizes adjacent to the HCF. In addition, EVs of different sizes were distributed throughout the matrix (Fig. 1C, arrowheads) and just inside the cell membrane (Fig. 1D, white arrows), whether they are exosomes or endosomes is unknown. It is not clear if the variation in size represents different EVs or just the same EVs but of different sizes.

**Figure 2:**
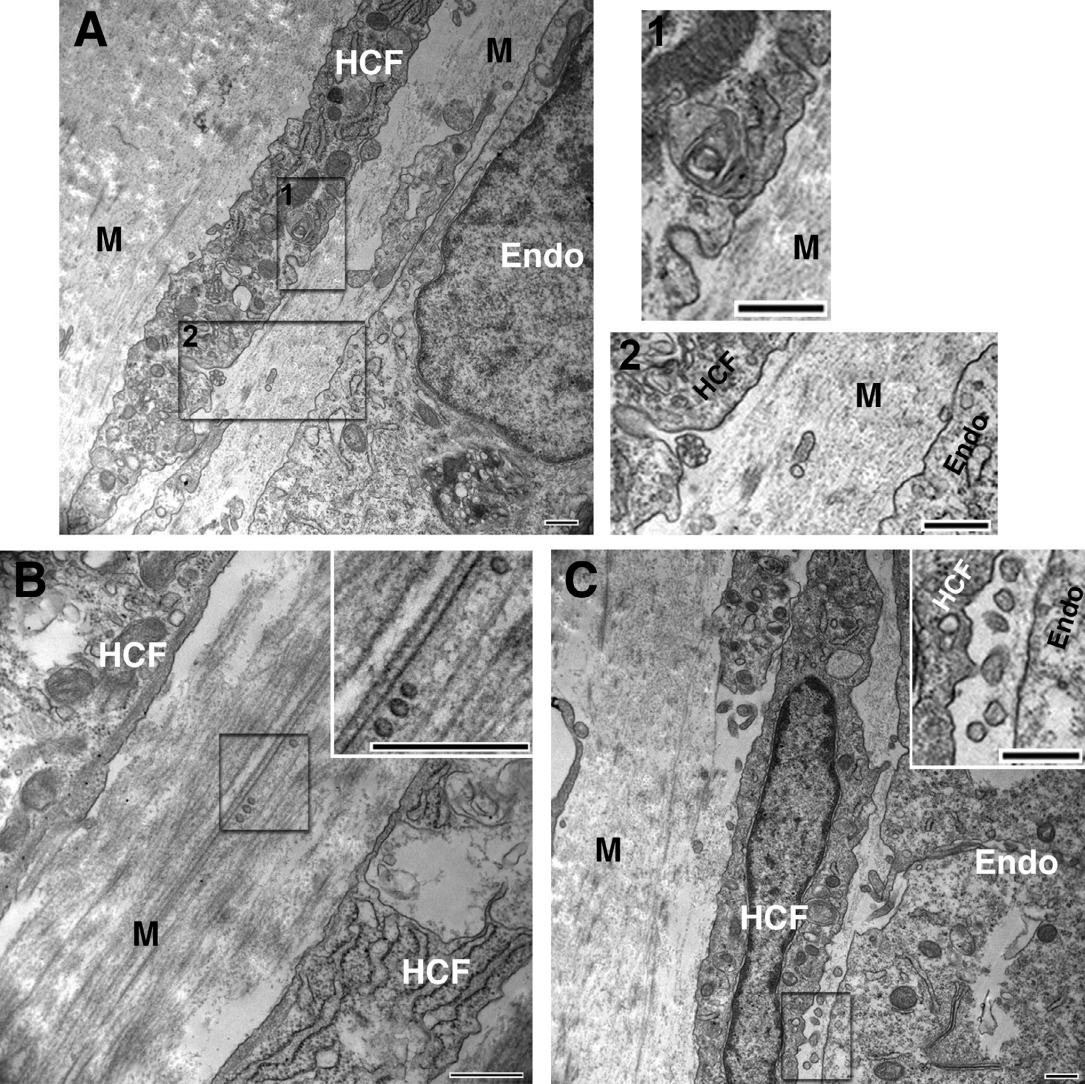
Transmission electron micrographs demonstrating EVs in coculture of HCF and endothelial cells. (**A**) Large pits were observed in HCF, which are outlined and magnified (A_1,2_). (A_2_) Note the bundle of EVs either entering or exiting the cell through a large pit. (**B**) EVs observed within the matrix (outlined). Inset shows higher magnification of outlined area. (**C**) EVs observed between HCF and endothelial cell (outlined). Inset shows higher magnification of outlined area. HCF, human corneal fibroblast; Endo, human endothelial cell; M, matrix; Bars = 500 nm.

To examine the potential for cell–cell interaction between different types of corneal cells, we generated 3D cocultures consisting of HCF in a self‐assembled matrix and primary human corneal endothelial cells (Fig. 2). As with the HCF construct (Fig. 1), numerous EVs were present in the cocultures (Fig. 2). Interestingly, large pits were formed in the HCF (Fig. 2A_1,2_), which appear to provide an entry or exit port for EVs into or out of the cell, and a bundle of EVs was observed entering/exiting the cell through one of these pits (Fig. 2A_2_). A pit was also present in the HCF construct (Fig. 1A,*). In addition, we observed that EVs migrated through the matrix and were often found within the extracellular matrix, distant from the cells (Fig. 2B, inset). Of great interest was the impressive number of EVs present between endothelial cells and HCF, suggesting the possibility of cell–cell communication between cell types (Fig. 2C, inset). It should be noted that it is possible that some of the structures may be cross‐section of cellular processes.

**Figure 3:**
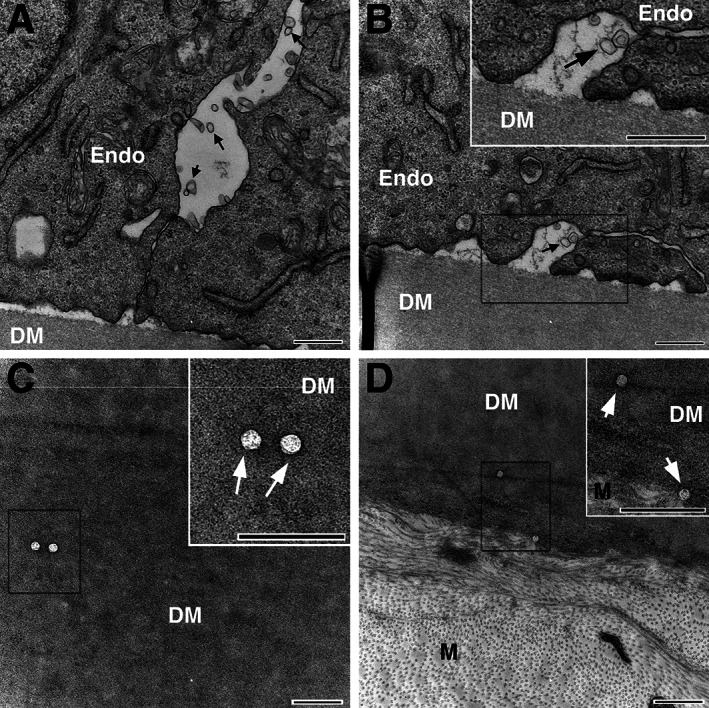
Transmission electron micrographs demonstrating EVs in *ex vivo* rabbit cornea. (**A**) EVs were present between cells (arrows). (**B**) EVs observed between endothelial cell and Descemet's membrane (outlined). (**C**) EVs appeared midway through Descemet's membrane (outlined). (**D**) EVs seen either exiting or entering Descemet's membrane (outlined). Insets show higher magnification of outlined areas (B–D); arrows indicate EVs. Endo, rabbit corneal endothelium; DM, Descemet's membrane; M, matrix; Bars = 500 nm.

Finally, we examined EVs in intact rabbit corneas. Numerous EVs were present between endothelial cells (Fig. 3A, arrows), as well as between endothelial cells and Descemet's membrane (Fig. 3B, arrow). In Figure 3C, EVs appeared to have penetrated into the Descemet's membrane, and in Figure 3D, extracellular vesicles were observed to be exiting/entering the Descemet's membrane to/from the stroma, suggesting that endothelial cells and HCF can potentially communicate with one another via EVs.

## DISCUSSION

Cell–cell communication is an important component of most corneal functions, including fibrosis, regeneration, and homeostasis, and its importance has been known for many years, especially in wound repair, as demonstrated by findings that stromal cell death resulted after epithelial removal (Wilson et al., 1996) and ulceration was prevented after blocking epithelial migration over a wound (Kenyon et al., 1979). Numerous soluble growth factors and cytokines have been examined for their role in corneal wound repair; however, an underlying question remained, “How do these factors migrate from one cell to another?” One potential answer to this question involves the release and uptake of EVs by adjacent cells, which can influence many of the functions in recipient cells (Di Rocco et al., 2016; Dos Anjos Pultz et al., 2017; French et al., 2017; Gopal et al., 2017; Kahlert and Kalluri, 2013). Extracellular vesicles were originally thought to be a method to remove cell debris; however, they have since been demonstrated to regulate many physiological (Di Rocco et al., 2016; Dignat‐George and Boulanger, 2011; Dos Anjos Pultz et al., 2017; Gopal et al., 2017; Kahlert and Kalluri, 2013; Puddu et al., 2010; Quesenberry et al., 2015) and pathological processes, including cancer. Over recent years, the role of EVs in cancer has been extensively studied and most components of cancer progression involve EVs, including inflammatory response, angiogenesis, metastasis, cell migration and proliferation, and immune suppression (Dos Anjos Pultz et al., 2017; Gopal et al., 2017; Kahlert and Kalluri, 2013). Cancer cells control these mechanisms by releasing EVs that act on the host's cells to produce a microenvironment that promotes recruitment and growth of cancer cells. This occurs in both the primary tumor, where EVs travel relatively short distances, and in metastasis, where the EVs travel long distances. We believe that this also may be the case with signaling for corneal wound healing, that the wounded epithelium releases EVs with certain signaling components into the stroma, and these EVs are picked up by the stromal keratocytes, which then respond to the signal.

To show that EVs are involved in signaling for corneal wound healing, we first needed to prove that EVs were present in the cornea. This was accomplished by Han et al. (2017), who showed that EVs could be isolated from both human epithelial and stromal cells in culture. In the present study, we took the next step and examined if EVs could be observed in a more natural 3D environment. As seen in Figures 1, 2, 3, we were able to visualize EVs in our 3D construct and coculture, as well as in an *ex vivo* rabbit cornea. Extracellular vesicles were found to be associated with cells (Figs. 1; 2A,C; 3A,B), within the matrix (Figs. 1C; 2B), and within the Descemet's membrane (Fig. 3C,D).

During the course of this study, numerous intriguing observations were made. The first being the large number of EVs present between the HCF and primary human corneal endothelial cells (Fig. 2), suggesting that there is indeed potential for cell–cell communication between these two cell types via EVs. The second being the large pits observed in the HCF (Figs. 1A; 2A). These pits could potentially be entry ports for the EVs, and is one of many mechanisms that have been proposed for EV entry into cells. A third interesting observation was EVs alone within the matrix away from cells (Fig. 2B), thus indicating that EVs travel further than just to adjacent cells within the cornea; however, it is unknown whether the EVs are moving to a site or simply moving at random. Finally, EVs are unable to penetrate the corneal basement membrane, which agrees with multiple publications that once the basement membrane is reformed and barrier function restored, topical applications of therapeutics are unable to affect the stroma (Medeiros et al., 2018); however, in this study, EVs appear to penetrate Descemet's membrane (Fig. 3C,D). This final fascinating observation suggests that the anterior and posterior HCF may be subject to different degrees of cell–cell communication *in vivo*, thus supporting a number of anecdotal suggestions that anterior and posterior HCF exhibit different characteristics. Taken together, this data suggest that the 3D‐culture system both *in vitro* and *ex vivo* will allow us to examine EVs in a more natural environment, determine if EVs do indeed provide the cell–cell communication between the different corneal cell types, and observe how EVs reenter corneal cells.

An exciting aspect of EVs is their potential to be used as a method to deliver therapeutics. Extracellular vesicles are known to travel long distances through the blood stream and to have some specificity for particular tissues, and since they are lipid derived, they also pass through tissues (Ha et al., 2016; Sutaria et al., 2017). Particularly exciting is that it is fairly easy to “load” EVs with drugs or proteins of interest. For small molecules, EVs can be loaded simply by incubating them with the cargo. For larger molecules, a variety of methods to create pores in the EVs have been developed. Importantly, once the treatment is removed, the EVs return to their original form (Ha et al., 2016; Sutaria et al., 2017). Examples of the use of EVs as a delivery system include the use of curcumin (Ha et al., 2016), catalase (Haney et al., 2015), as well as mRNA and miRNA (Ha et al., 2016). In the catalase study, the loaded EVs were introduced into the blood stream by IV injection. Excitingly, the loaded EVs were transported across the blood–brain barrier. In the study with curcumin (Ha et al., 2016), curcurmin was added to a solution of EVs, purified, and also injected by IV. The treatment was found to lower inflammatory cytokines, such as interleukin‐6 and tumor necrosis factor‐alpha. One potential problem with IV injection of EVs is that they are removed from the circulation within minutes by the host's macrophages in the liver and spleen (Bala et al., 2015; Yanez‐Mo et al., 2015). One method to overcome this is to use topical application. Extracellular vesicles could be loaded with different wound inhibiting or healing proteins or genes (i.e., TGF‐ß1 or ‐ß3) and then applied directly to the tissue, thus eliminating the dilution of the EVs. From our studies, the 3D coculture system would provide an excellent model to study the use of topical EVs for therapy.

In summary, EVs appear to have the potential to be involved in many of the mechanisms in the cornea, and the 3D coculture system provides an *in vivo*‐like model to examine these interactions. In addition to corneal epithelial, fibroblast, and endothelial cells, nerves and inflammatory cells also release EVs. The 3D coculture system eliminates the effect of the inflammatory cells and the nerves; however, EVs from these cells can be added to the 3D model in a controlled manner in order to examine how nerves affect cells that they do not innervate, and inflammatory cells affect cells in a wounded or diseased state. The study of EVs appears to be on the verge of a revolution.
